# Phytochemical standardization, formulation and evaluation of oral hard gelatin capsules from *Pinus eldarica *bark extract

**Published:** 2021

**Authors:** Sajad Esmaeili, Ladan Dayani, Azade Taheri, Behzad Zolfaghari

**Affiliations:** 1 *Department of Pharmacognosy, Faculty of Pharmacy and Pharmaceutical Sciences, Isfahan University of Medical Sciences, Isfahan, Iran*; 2 *Department of Pharmaceutics, Faculty of Pharmacy and Pharmaceutical Sciences, Isfahan University of Medical Sciences, Isfahan, Iran*

**Keywords:** Pinus eldarica, Capsules, Plant extracts, Phytochemical, Standardization

## Abstract

**Objective:**

The extract of *Pinus eldarica* bark contains many polyphenolic compounds that were studied due to their high antioxidant, anti-inflammatory and anti-mutagenic effects. Therefore, the purpose of the present study was to conduct phytochemical standardization and develop hard gelatin capsules from the extract of *P. eldarica* bark.

**Materials and Methods:**

Extraction was carried out by maceration method at room temperature for 72 hr using ethanol 70% followed by freeze drying. Quantification and standardization tests were performed using Folin-Ciocalteu method. Then, nine formulations were prepared containing different amounts of stearic acid (1-3%) and corn starch (3%, 10%, and 25%). Each formulation was characterized by FTIR and pharmacopoeial tests such as drug content, disintegration time, flowability parameters and drug release percent. The optimized formulation underwent stability studies at 75±5% humidity and 40±2°C.

**Results:**

The total phenolic content of the extract in terms of gallic acid equivalent was 362.8±5.4 mg/g and the total procyanidin content in the extract was 174.386±2.5 mg/g. FTIR revealed no interaction between the components. The results presented that the best formulation of the capsules was achieved they contained 3% of stearic acid and 25% of corn starch. This formulation showed 91.69±0.33% of drug content, 9.36±0.02 min disintegration time and 83.02±0.81% release percent. Moreover, it showed good flowability. Stability studies on the optimized formulation displayed that the formulation was stable within 6 months in the accelerated condition.

**Conclusion:**

In conclusion, results of the present phytopharmaceutical evaluations confirmed this product as a promising herbal capsule formulation.

## Introduction

Nowadays, herbal medicines are considered an attractive choice for treatment of various types of diseases. Pycnogenol^®^ (PYC) is the registered trade name for a French formula of a specific blend of procyanidins extracted from the bark of the *Pinus maritime* or *Pinus pinaster* (Sarvmeili et al., 2016 ▶). PYC is commercially available as a herbal dietary supplement in tablet or capsule dosage form at doses between 20 to 100 mg. It is used worldwide as an over-the-counter product for numerous diseases ranging from chronic inflammation to circulatory dysfunction. Reviews of the extract highlighted its antioxidative nature and strong free radical-scavenging activity against reactive oxygen and nitrogen species (Simpson et al., 2019 ▶). It was described that the extract is considered one of the best nutraceutical agents in this regard, as well. Tremendous antioxidant capacity of PYC bark extract may be in part, due to appreciable amounts of polyphenolic compounds. Also, it contains 65-75% procyanidins (Rohdewald, 2002 ▶). Studies have shown that PYC displays greater biological effects as a mixture than its purified components. Moreover, the components interact synergistically and it is classified as generally recognized as safe (GRAS) according to the clinical safety and preclinical toxicology data (Rohdewald, 2015 ▶, Sarvmeili et al., 2016 ▶).

*Pinus eldarica *Medw, called Iranian pine or Tehran pine, belongs to Pinaceae family. It is an evergreen tree that grows natively in Iran, Afghanistan and Pakistan. Various parts of Tehran pine have been widely used in traditional medicine for treatment of different diseases such as wound infections, bronchial asthma, skin irritations, allergic rashes and dermatitis (Faridi et al., 2012 ▶, Ghadirkhomi et al., 2016 ▶). Bolandghamat et al. (2011) ▶ demonstrated that ethanolic extract of *P. eldarica *Medw needles at a dosage of 300 mg/kg has an antidepressant effect in Wistarrats. In another study, it was shown that oral administration of the hydroalcoholic extract of *P. eldarica* bark at the doses of 125 and 250 mg/kg, considered relatively non-toxic in Wistar rats (Ghadirkhomi et al., 2016 ▶). Moreover, *P. eldarica* nut reduces blood cholesterol level and aortic atherosclerotic participation in hypercholesterolemic rabbits (Huseini et al., 2015 ▶). Its fruit extract prevents calcium oxalate deposition, without producing diuresis (Hosseinzadeh et al., 2010 ▶). Besides, Iravani and Zolfaghari (2014) ▶ reported that *P. eldarica* bark extracts can be used as an effective source of polyphenolic compounds in food and pharmaceutical industries. High level of reactive oxygen species (ROS) is strongly linked with pathogenesis of many disorders so inactivation of superoxide and hydroxyl radicals is among the most important beneficial effects of the compounds. Because antioxidant activity can prevent accumulation of oxidatively damaged proteins and may reduce the risk of several neurodegenerative diseases such as Parkinson's, Alzheimer's, and Huntington's diseases (Iravani and Zolfaghari, 2011 ▶, Tayarani-Najaran, 2019 ▶). In addition, these compounds show cytotoxic activity which can be useful for anticancer effect and inhibit oxidative hemolysis, lipid peroxidation, etc. (Iravani and Zolfaghari, 2011 ▶). According to the phytochemical studies, that the herbal extract possessing high amounts of phenolic compounds, revealed strong antioxidant activity which especially include catechin, taxifolinand other phenolic acids such as caffeic acid and ferulic acid showed anti-inflammatory and anti-mutagenic effects (Afsharypuor and Sanaty, 2005 ▶, Babaee et al., 2016 ▶, Iravani and Zolfaghari, 2013 ▶, Iravani and Zolfaghari, 2014 ▶, Sarvmeili et al., 2016 ▶). It is worth mentioning that polyphenolic compounds were found both in *P. pinaster* and *P. eldarica* bark (Sarvmeili et al., 2016 ▶). So, it can be speculated that *P. eldarica* bark extract in capsule or tablet dosage form, may be considered an alternative for Pycnogenol^®^ capsules. 

Therefore, the present study was designed to prepare an optimized formulation of hard gelatin capsules from *P. eldarica* bark extract.

## Materials and Methods

**Ethical considerations**

The Ethics Committee of Isfahan University of Medical Science approved this research project with the Ethics ID IR.MUI.RESEARCH.1398.494.

**Chemicals and reagents**

Ethanol, methanol, butanol, hydrochloric acid, ferric ammonium sulfate, gallic acid and sodium carbonate were prepared from Merck Company (Germany). Microcrystalline celloluse (MCC), Stearic acid, Corn starch, Colloidal Silicon Dioxide (Aerosil^‏^^®^ 200) and Folin-Ciocalteu reagents were purchased from Farabi Pharmaceutical Industry Company (Iran). *P. eldarica* barks from the trunk of the tree, were collected from Isfahan province inOctober 2018. The pine samples were authenticated by Mahboubeh Khatamsaz and the herbarium was prepared under the acquisition number of 3318 in the Pharmacognosy Laboratory of the Department of Pharmacognosy in the School of Pharmacy and Pharmaceutical Sciences.

**Pine barks samples**

*P. eldarica* barks were washed and air-dried under shading at room temperature (25±2°C). The bark of the tree was powdered using a conventional grinder to produce uniform particles. 

**Extraction of the plant material**

The air-dried powder of the plant material (1 kg) was extracted thrice using 5 l of ethanol 70% at room temperature using the maceration process for 72 hr with occasional shaking; then, the mixture was filtered usinga Buchner funnel through Whatman^®^ cellulose filter paper 41. After that, the filtrate was subjected to the rotary evaporator (Bibby RE200, UK) at 40 to 50^°^C prior to freeze-drying, in order to remove the solvents and finally freeze-dried. The powder extract was stored in a closed container until further studies (Sadeghi et al., 2016 ▶). 

**Phytochemical screening of ethanolic extracts**

**Quantification of total phenolic content in** ***P. eldarica*** **bark extract**

Folin-Ciocalteu’s reagent method was employed to determine the total phenolic content which is also called the gallic acid equivalence (GAE) method (Singleton et al., 1999 ▶). Here, 20 ml of each sample was taken and well mixed with1.58 ml of distilled water and 300 μl of sodium carbonate 20% for 5 min. After that, the mixture was treated with 100 μl of diluted Folin-Ciocalteu reagent. After 2 hr in which the samples kept away from direct sunlight, the absorbance was measured at 765 nm using a double beam UV-VIS spectrophotometer (Bio-Tek, PowerWave XS, USA). To prepare a calibrated curve, various concentrations of gallic acid (50, 100, 150, 250, and 500 mg/l) were made and the absorption-concentration curve was plotted. The total phenol content was estimated using a standard curve and expressed as mg/g of GAE by the following equation:

 TPC = CV / m 

Equation 1

In which, TPC is total phenolic content, C is the concentration obtained from the calibration linear equation, V is the volume and m is the weight of the extract (Waterhouse, 2002 ▶). 

**Determination of procyanidins in** ***P. eldarica*** **barks extract**

One milliliter of sample solution and 1 ml of methanol were transferred into two separate test tubes. To each test tube, 6 ml of reagent A and 0.25 ml of reagent B were added. Both tubes were mixed and heated in a water bath at 60^°^C for 45 min, then, cooled rapidly in an ice bath. The color changes in the sample solution (orange-red) compared to the control (yellow) indicated the presence of procyanidins. After that, each of these samples were transferred to 10 ml vials separately and quantified using reagent A. The solution containing methanol was used as the blank. The absorbance of the sample solution was measured by UV spectrophotometery at λ_max_=546 nm. The amount of total procyanidin in the extract was determined by the following formula: 

 (2000Au)/(36.7W) 

Equation 2

Au is the absorbance of the sample, 36.7 is pine procyanidin absorption coefficient, and W is the weight of Eldarica Pine taken to prepare the Sample stock solution (mg).

Reagent A: Butanol and hydrochloric acid (95:5).

Reagent B: 2 g of ferric ammonium sulfate in a mixture of 100 ml of water and 17.5 ml of hydrochloric acid.

**Preformulation parameters**

**Bulk density and tapped density and Carr’s index**

From the extract powder or the formulations displayed in Table 1, 7 g was passed through a sieve with a mesh number of 18 in order to break up the agglomerates. Then, poured in to the graduated cylinder (readable to 2 ml) and bulk volume was measured (V_0_). Tapped volume (V_f_) was attained after 100 times of tapping the cylinder from a height of 14±2 mm. The compressibility index or Carr’s index and Hausner’s ratio were calculated using the following equations:

$\mathrm{Compressibility Index}=100\times \left[\frac{V_{0}-V_{f}}{V_{0}}\right]$

$\mathrm{Hausner Ratio}=\frac{V_{0}}{V_{f}}$

equation 3 and equation 4

**Angle of repose**

A weighed quantity (7g) of powdered materials was passed slowly through the funnel in which the height was maintained approximately 2–4 cm from the top of the powder pile by a stand. When the whole sample poured out of the funnel, the height and diameter of the base were noted and angle of repose was calculated by the formula stated below:

tanα=height0.5 base

equation 5

**Formulation and evaluation of hard gelatin capsules**

To prepare the capsules from *P. eldarica* bark extract, first, nine different formulations (shown in Table 1) were mixed well and finally sifted through sieve No. 18. Gelatin capsules (size elongated zero) were used to encapsulate the powders of nine different formulations (Table 1) using a hand-held capsule filling system. Each formulation contained 200 mg of the extract and total weight of each capsule was adjusted to350 mg by MCC as diluent and disintegrants. The variables in the formulations set as the percent of corn starch as disintegrating agent (3%, 10%, 25% w/w of the whole formulation weight) and stearic acid as lubricant (1-3% w/w of the whole formulation weight). At last, the capsules were evaluated for the following physicochemical characteristics (Vyas et al., 2011 ▶).

**Table 1:** Composition of different formulations of *Pinus* *eldarica* capsules

**Formulation code**									
F9	F8	F7	F6	F5	F4	F3	F2	F1	**Ingredients (mg)**
200	200	200	200	200	200	200	200	200	**Extract**
87.5 (25%)	35 (10%)	10.5 (3%)	87.5 (25%)	35 (10%)	10.5 (3%)	87.5 (25%)	35 (10%)	10.5 (3%)	**Corn starch**
10.5 (3%)	10.5 (3%)	10.5 (3%)	7 (2%)	7 (2%)	7 (2%)	3.5 (1%)	3.5 (1%)	3.5 (1%)	**Stearic acid**
1	1	1	1	1	1	1	1	1	**Colloidal silicon dioxide**
51	103.5	128	54.5	107	131.5	58	110.5	135	**MCC**
350	350	350	350	350	350	350	350	350	**Total weight**

**Estimation of drug content**

Here, Folin-Ciocalteu method was used. Ten capsules of each formulation were carefully opened and the content was completely removed. Then, the contents were mixed well and weight of powder equivalent to one capsule was determined. Next, 10 ml ethanol was added to the chosen powder and after extraction, the suspension passed through a paper filter. The Folin-Ciocalteu method was performed and the absorbance was measured at 765 nm.

**Weight variation test**

First, 10 capsules were selected randomly. Second, each capsule was weighed accurately on an analytical balance (M1, M2, ...) and the average weight of the capsules was calculated (W). A is the content of drug substance (in percentage) obtained using UV spectroscopy. Then, using the results of 3 time assay of each formulation, the amount of active ingredient in each capsule was calculated in percentage (X1, X2, …) using the following equation: 

Xi=Wi × A/W equation 6

Then, the mean amount of active ingredient in capsules (X) and standard deviations (SD) for X1, X2, ..., were calculated. Finally the Acceptance value (AV) was obtained by using the USP formulas (Detailed procedure is ascribed in the USP 42 <905>)**.**

***In vitro*** **drug release percent determination**

For the purpose of determination of the drug release percent (%Rel), a Pharma Test^®^ rotary paddle dissolution test apparatus (Apparatus 2) was used. The release medium temperature was set at 37±2^°^C and the pedal rotation speed was fixed at 50 rpm. Each vessel was filled with 900 ml of phosphate buffered saline (pH 6.8) for %Rel test process. After reaching the desired temperature, the capsules were placed in the vessels and sampling was performed from the site as specified by the pharmacopoeia within 25 min after the pedals were rotated. The sample was filtered through a 0.45 µm syringe filter and then by the Folin-Ciocalteu method, the amount of active ingredient released was determined spectrophotometrically.

**Disintegration test**

*In vitro* disintegration time of the capsules was determined by the disintegration tester (Pharma Test^®^ PTZ-E, Germany) in accordance with the United States Pharmacopoeia method <701>. The bath was filled with distilled water and the temperature was set at 37±2^°^C. One capsule was put into each of the six tubes. The basket in the fluid was going up and down for 29 to 32 times per minute so that the metal mesh was always below the liquid surface during the test. The apparatus operated until all six capsules were disintegrated leaving only remnants of gelatin shell on the mesh.

**Fourier transforms infrared spectroscopy (FT-IR)**

The physical mixture of sieved fractions of pine bark extract and excipients was prepared by mixing all components of the optimized formulation using a mortar and pestle and then, kept in an oven with 75±5% relative humidity (RH) at40±2^°^C for two weeks. The FTIR spectra of the pine bark extract and the physical mixture of excipients with the pine bark extract were obtained using Rayleigh^®^ WQF-510. Samples were mixed with potassium bromide (KBr) then pressed to form a disk and scanned against a blank KBr disk at wave lengths ranging from 4000-650 cm^-1^. 

**Stability study**

The experiment was conducted according to the International Council for Harmonisation (ICH) guidelines in an accelerated condition with75±5% RHat40±2^°^C for a period of 6 months. Adequate numbers of capsules filled with the optimized formulation, were placed in a chamber for 6 months. Required capsules were taken after one, three and six months in triplicate for analysis. The stability in disintegration time, drug content and %Rel of the capsules was investigated (Bankoti, 2012 ▶).

**Statistical analysis**

Statistical analysis was performed by SPSS, version 20.0, SPSS Inc., using one-way analysis of variance (ANOVA) followed by Duncan *post-hoc* test for multiple comparisons. P values less than 0.05 were considered statistically significant. The data is reported as mean±SD.

## Results

The yield of freeze-dried alcoholic extract obtained from *P. eldarica* barks on average, was 21.23±1.03%.

**Total phenol and procyanidin content in the extract**

**Figure 1:**
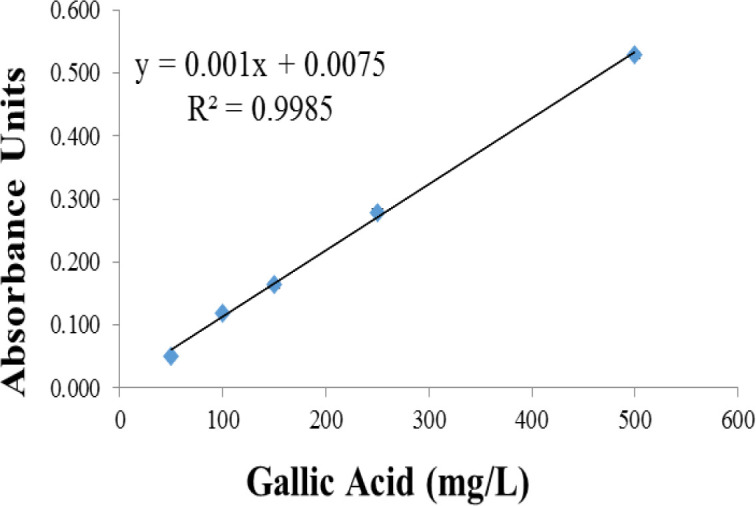
Standard curve of gallic acid

Figure 1 shows the calibration curve for gallic acid. As observed, it showed good linearity with Regression coefficient of 0.9985 at the concentrations of50-500 μg/ml. The total phenolic content was 362.8±5.4 mg GAE/g extract while the total procyanidin content was 174.386±2.5 mg/g of extract based on eq 2.

**FTIR testing**

**Figure 2:**
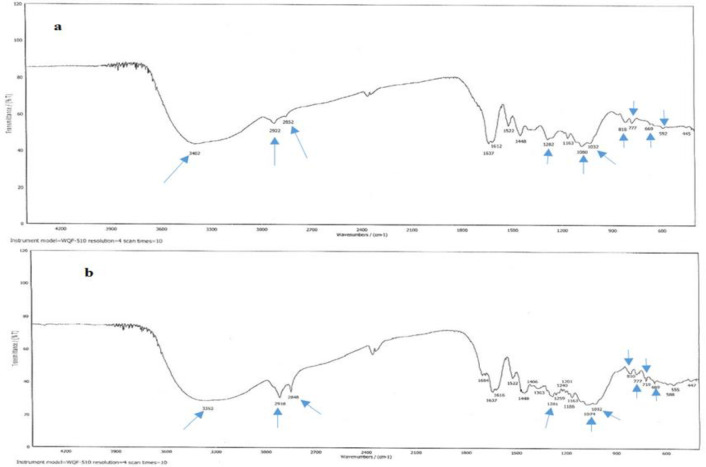
The FTIR spectroscopy results of a) pine bark extract powder b) physical mixture of all excipients with pine bark extract powder

As seen in Figure 2, the characteristic absorption bonds were quite similar to the primary pine bark extract. The peak at 3352 to 3402 cm^-1^ is assigned to –OH stretch vibration in phenolic and aliphatic structures. Small peaks at 2922 and 2852 cm^-1^ originate from –CH stretch vibration in aromatic methoxyl groups and in methyl and methylene groups of side chains. The peaks between 1400 and 2000 cm^-1^ show the aromatic nature of the structure. The bond at 1370–1380 cm^-1^ is attributed to phenolic stretch vibration of –OH and aliphatic –CH deformation in methyl groups. Aromatic –CH bending in plane bending vibration is detected at 1080 cm^-1^ and a –CO stretch vibration is produced at 1032 cm^-1^. For peaks at wavelengths smaller than 900 cm^-1^, aromatic –CH stretch vibration is detected. 

**Physicochemical characteristics of capsules**

The results of physicochemical analysis of different studied formulations, are presented in Table 2.

**Drug content**

All capsule formulations pass the test for assay as all of them were within 85-115% (Table 2). 

**Weight variation**

The requirements for dosage uniformity of the capsules were met if the acceptance value (AV) of the 10 dosage units is less than or equal to L1% which is considered 15. The prepared capsules showed good weight uniformity according to the USP 42 because the AVs were all less than 15 (Table 3).

**The flowability properties of the formulations**

As inferred from Table 2, Hausner's ratio was in the range of 1.05-1.21. All formulations showed a ratio below 1.35 which means that all of them depicted good flowability. The minimum range of the ratio belongs to the last three formulations which had3% of stearic acid. Based on Table 2, the compressibility indices of the formulations were in the range of 7.86-19.45%. According to the statistical analysis, by increasing the stearic acid as lubricant, the fall in the compressibility indices was considerable (p<0.05). Also, all of compressibility indices were below 25%. Table 2 indicates the angle of repose for all formulations. As seen, all formulations gave an angle of repose in the range of 27.4-37.9°. Moreover, F9 showed significant difference in comparison to the others (p<0.05).

**Disintegration time**

The formulation of the capsule complies with the test according to the USP, if all of the capsules have disintegrated completely within 30 min (Uddin et al., 2016 ▶). The disintegration time of various capsules was between 9.36±0.02 and20.03±0.03 min (Table 2). As seen in Table 2, F7, F8 and F9 showed the lowest time of disintegration which were significantly different from other formulations (p<0.05).

**Drug release percent**

The F3 capsules with 3% starch, showed the lowest release percent (%75.15±1.32) (Table 2). Drug release study of *P. eldarica* capsules indicated that the percentage release of API for F9 was 83.02±0.81% after 25 min which revealed the highest release among all. 

**Stability test**

F9 was chosen for further studies. The accelerated stability study of capsules was done according to ICH guidelines during 6 months. Observations are shown in Table 4. 

**Table 2:** Results of determination of the active ingredient content, disintegration time, and compressibility index in different formulations. Values are expressed as mean±SD (n=3)

**Formulation Code**	**Drug content (%)±SD**	**Disintegration time (min)±SD**	**Compressibility index (%)±SD**	**Angle of repose** **(Degree)±SD**	**Hausner's ratio±SD**	**Drug release** **% in 25 minute** **±SD**
F1	90.66±1.09	20.03±0.03	19.51±0.05	33.39±0.06	1.24±0.04	75.76±2.68
F2	86.75±0.90	19.11±0.01	13.69±0.06	37.83±0.08	1.15±0.05	75.50±2.03
F3	87.39±1.68	16.30±0.01	14.28±0.09	33.80±0.08	1.16±0.08	75.15±1.32
F4	86.90±0.77	19.25±0.02	18.18±0.11	33.88±0.06	1.22±0.10	78.34±0.88
F5	88.29±0.79	12.04±0.03	18.18±0.07	35.36±0.12	1.22±0.07	78.72±0.95
F6	91.20±0.49	16.14±0.03	14.28±0.09	37.17±0.05	1.16±0.08	79.42±1.64
F7	91.36±0.82	13.60±0.07	9.09±0.05	29.65±0.10	1.10±0.04	81.91±1.07
F8	91.53±0.45	9.51±0.05	7.93±0.06	29.20±0.08	1.08±0.06	82.51±0.81
F9	91.69±0.33	9.36±0.02	7.93±0.04	27.43±0.06	1.08±0.04	83.02±0.81

**Table 3:** Results of measuring the uniformity of the active ingredient in the capsules (AV=acceptance value)

	**F1**	**F2**	**F3**	**F4**	**F5**	**F6**	**F7**	**F8**	**F9**
AV	9.85	10.80	11.53	14.98	12.57	11.13	12.25	12.51	2.43

**Table 4:** Results of stability test on optimized capsules

**Time (month)**				**Limitations**	**Test**	**No.**
6	3	1	0			
9.7	9.9	9.8	10.0	NMT- 30 minutes	Disintegration time	**1**
82.2	82.5	83.2	82.7	Over 70% release	%Rel test	**2**
179.5	179.8	181.4	183.1	170-230	Assay:(mg/Cap( Extract	**3**

The stability parameters were analyzed after 0, 1, 3 and 6 months of storage at accelerated conditions of temperature, and relative humidity. It was indicated that different characteristics did not produce any significant changes (p>0.05). The content of *P. eldarica* extract was 183.1, 181.4, 179.8 and 179.5 mg GAE/g after 0, 1, 3, and 6 months respectively as shown in Table 4. Hence, disintegration time and %Rel did not undergo significant changes (p>0.05). The results indicated that the F9 formulation is stable in the prescribed storage conditions.

## Discussion

In this research, the maceration method was used by ethanol 70% for three days and then, the extract was freeze-dried to produce the powder form. The yield of freeze-dried alcoholic extract obtained from *P. eldarica* barks on average was 21.23%. This is in accordance with previous studies where 21 or 20% was reported (Babaee et al., 2016 ▶, Ghadirkhomi et al., 2016 ▶). 

When Pine bark extract is changed to the powder form, it is characterized as a brown powder that is stable in dark and dry place and is highly soluble in aqueous medium and absorbed rapidly after oral administration (Maimoona et al., 2011 ▶). The preliminary phytochemical screening of the *P. eldarica* bark extract verified the presence of polyphenolic compounds (PC). The present study indicated that the extract had 362.8±5.4 mg GAE/g of PC equal to 36.28%. Previous studies also reported that the bark extract of *P.eldarica *has high amounts of PC compared to the other parts (Babaee et al., 2016 ▶). Ghadirkhomi et al also described 38.1±1.5% of GAE in the extract of *P. eldarica *bark (Ghadirkhomi et al., 2016 ▶). In another study, 37.04±1.8% of GAE in dried barks of *P. eldarica *was estimated (Babaee et al., 2016 ▶). Hence, it was found that 17.44% by weight of extract powder is procyanidins; in other words, 48% by weight of polyphenols was procyanidins.

Drug-excipients compatibility assessments provide rational basis for selecting excipients used to design a formulation. In FTIR spectroscopy by analyzing significant changes in the shape and position of the absorbance, the interactions between the ingredients could be implied. If there is bond shift or broadening in the peaks referring to the functional groups compared to the spectrum of the API, there is an interaction between active drug and excipients (Prathyusha and TEGK, 2013 ▶). In accordance with the FTIR spectra (Figure 2), the results of the analysis of the formulation components showed that no new bonds or broadening were formed and thus, no incompatibility or interference between the constituents existed. 

In this research, to prepare capsule formulations, MCC as filler or diluent and disintegrant, corn starch as disintegrating agent, stearic acid as lubricant and colloidal silicon dioxide (Aerosil^®^ 200) as glidant was used and the elongated zero size was taken.

Determination of drug contents is very important in calculating the percentage released and ensuring uniform pharmacological response among different batches (Uddin et al., 2016 ▶). The results associated to the percent of API in the capsules tabulated in Table 2, indicated that all the formulations had active ingredient in the range of 85-115% and F9 showed the highest percent.

To ensure the consistency of dosage units, each unit in a batch should have drug substance content within a narrow range around the label claim. Table 3 shows that AV was within 2-14 for all nine capsules and fulfilled the requirement of standard content uniformity because the AVs were all less than 15 but the F9 formulation had less content uniformity compared to other formulations. This means that the method of filling the capsules was satisfactory and reflected low variations in the content of the active ingredient.

To examine the flowability of the formulations based on the USP general chapter <1174>, since powder behavior is multifaceted and thus complicates the effort to characterize powder flow, various tests can be executed to determine Hausner's ratio, compressibility index and angle of repose. According to the data pertaining to the flowability parameters in Table 2, with the increments of the lubricant, the flow properties got better and F9 showed best flowability than other formulations and the difference was considerable (p<0.05). Hence the data shows that the F9 significantly has least Hausner's ratios, compressibility indices and angles of repose (p<0.05). Good flow of powder helps to avoid the extensive costs and time wasting and achieve the best formulation and improve the quality and consistency of the product (USP 42 NF37, 2019).

Excipients which could affect the drug dissolution and disintegration, could in turn influence the speed of absorption and the bioavailability (Carvalho et al., 2013 ▶). Hence, selecting the best formulation of the design based on disintegration time is critical. Some of the components in the formulations such as disintegrants could facilitate disintegrating and thus, the dissolution rate. For hard gelatin capsules, USP 42 stated that at the end of 30 min, the herbal capsules passed the test as no residue of the drug was remained (USP 42 NF37, 2019). It was found that all formulations were disintegrated in less than 30 min (especially F8 and F9, were disintegrated significantly earlier than others(p<0.05)). Also, it seemed that by increasing the percentage of disintegrants (MCC & starch), the disintegration time was decreased (Table 2).

With the purpose of evaluating the dissolution percentage in different formulations, the dissolution test was carried out following the established methodology for United States pharmacopeia <711>. It seems likely that the %Rel in all capsules was related to the disintegration time. The releasing percent of capsules revealed that they were dissolved up to 75% within 25 min (Table 2). It is expected that the percent of stearic acid as lubricant has an indirect effect on the %Rel. So, by increasing the lubricant, the %Rel decreased. Nevertheless, this is in contrary to our results. Probably, since the percent of the stearic acid used in this experiment (1%, 2% and 3%) was lower than 5%, the effects of this hydrophobic excipient on %Rel were not significant. This could explain the results attained in our study.

Drug release in the capsules was rapid particularly in F8 and F9 because of the fast disintegration time. It could be noticed that nearly 80% of the extract in F8 and F9 was released after 25 min of the experiment indicating an acceptable %Rel for conventional dosage forms (Uddin et al., 2016 ▶). These two formulations both disintegrated within 9 min resulting in faster drug %Rel and absorption (Anbu and Samuel, 2018 ▶).

In view of the former results, F9 was selected as the optimum formulation to continue the studies for stability studies as it showed the best flowability, highest drug content, lowest disintegration time and highest %Rel.

Doing stability tests is an essential step in developing a dosage form because it provides proofs if the quality of the product changes over time under the influence of various environmental conditions or not. Besides, it is important to gain regulatory approval for commercialization. The manufacturers usually perform accelerated stability tests on all new pharmaceutical products as an integral part of the product development program. F9 formulation was subjected to accelerated stability testing (40±2°C and 75±5% RH) as per the WHO guidelines (WHO guidelines, 1996 ▶). The accelerated stability study of capsules containing extract of pine bark was done according to WHO protocol for 6 months. Observations are shown in Table 4 for stability parameters during the studied periods. 

Stability studies indicated that F9 remained unchanged during 6 months. Disintegration time and %Rel did not undergo significant changes (p>0.05). These values were reproducible even after more than three months. Based on current studies, it is estimated that maybe these herbal capsules are stable at room temperature for 2 years (Bankoti et al., 2012 ▶).

In conclusion, the findings suggested that the formulated herbal capsules of *P. eldarica *bark extract possessed characteristics within permitted range for conventional dosage forms according to the pharmacopoeial standards. F9 with 3% of stearic acid and 25% of corn starch passed all the parameters tested. Results showed that the capsules were stable under accelerated conditions for 6 months. Future prospects include animal studies and clinical trials of the finished product are necessary for the safety and efficacy recognition. Consequently, results obtained from the existing study by phytopharmaceutical evaluation, provide a promising herbal capsule formulation. 
